# The Synthesis of (Magnetic) Crosslinked Enzyme Aggregates With Laccase, Cellulase, β-Galactosidase and Transglutaminase

**DOI:** 10.3389/fbioe.2022.813919

**Published:** 2022-03-03

**Authors:** Gordana Hojnik Podrepšek, Željko Knez, Maja Leitgeb

**Affiliations:** ^1^ Laboratory for Separation Processes and Product Design, Faculty of Chemistry and Chemical Engineering, University of Maribor, Maribor, Slovenia; ^2^ Faculty of Medicine, University of Maribor, Maribor, Slovenia

**Keywords:** enzyme stability, immobilization, crosslinked enzyme aggregates, immobilization efficiency, enzyme activity

## Abstract

Immobilized enzymes have important aspects due to the fact that they possess higher stability, have the possibility to be easily removed from the reaction mixture, and are much easier to use when compared to free enzymes. In this research, the enzymes laccase, cellulase, β-galactosidase (β-gal), and transglutaminase (TGM) were immobilized by two different methods: crosslinked enzyme aggregates (CLEAs) and magnetic crosslinked enzyme aggregates (mCLEAs). The processes for CLEAs and mCLEAs preparation with different enzymes have been optimized, where the aim was to achieve the highest possible relative activity of the immobilized enzyme. The optimal conditions of the synthesis of CLEAs in mCLEAs are described, thus emphasizing the difference between the two types of immobilization based on different enzymes. This comparative study, which represents the synthesis of crosslinked enzyme aggregates using different enzymes, has not been performed so far. Moreover, the obtained activity of CLEAs and mCLEAs is presented, which is important for further use in different biocatalytic processes. Specifically, of a higher importance is the selection of enzymes involved in immobilization, as they belong to the three different most applicable enzymes (oxidoreductases, hydrolases, and transferases). The study confirmed that the resulting activity of the immobilized enzyme and the optimization of enzyme immobilization depended on the type of the enzyme. Moreover, the prepared CLEAs and mCLEAs were exposed to the supercritical carbon dioxide (scCO_2_) at different pressures to determine the effect of scCO_2_ on enzyme activity in immobilized form. Additionally, to demonstrate the reuse and stability of the immobilized enzyme, the stability and reusability tests of CLEAs and mCLEAs were performed. The catalytic performance of immobilized enzyme was tested, where the catalytic efficiency and long-term operational stability of mCLEAs were obviously superior to those of CLEAs. However, the higher activity observed for CLEAs compared to mCLEAs suggests a significant effect of magnetic nanoparticles in the stabilization of an enzyme crosslinked aggregate structure.

## 1 Introduction

**FIGURE 1:**
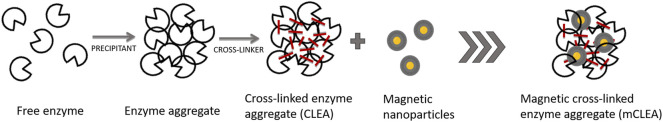
Preparation procedure of CLEAs and mCLEAs.

Enzymes, which accelerate biochemical reactions, have been extensively used in various fields due to their high catalytic efficiency and substrate specificity under mild reaction conditions (mild pH, temperature, and pressure). Nevertheless, their applications are severely limited by their poor stability and reusability properties (Liu and Dong, 2020). In this regard, immobilization techniques have been developed that allow the widespread use of enzymes. In general, there are many different types of enzyme immobilization such as adsorption, entrapment, encapsulation, covalent binding, and crosslinking, where generally physical and chemical methods for the immobilization of an enzyme are involved (Dwevedi, 2016). Depending on the type of interactions between enzymes and carriers, these techniques can be further classified into irreversible and reversible immobilization techniques. Covalent bonding, entrapment, and crosslinking are the most used procedures for irreversible immobilization of enzymes. Moreover, immobilization of enzyme by crosslinking refers to the process of immobilizing the enzyme *via* the formation of intramolecular and intermolecular crosslinks with specific groups of amino acids present on the surface of the solubilized enzyme (Chandra et al., 2020). Crosslinking can be achieved by adding bi- or multifunctional crosslinking reagents such as glutaraldehyde (GA) (Zang et al., 2020). This immobilization technique is usually carrier-free and involves assemblage of enzymes to form a three-dimensional structure (Luna et al., 2016). CLEAs synthesis is a promising strategy among other enzyme immobilization techniques, which also offers greater stability during operation and storage (Sulaiman et al., 2014) towards denaturation by heat, organic solvents, and autoproteolysis and are stable towards leaching in aqueous media (Sheldon, 2011). A high content of free amino groups, especially on the surface of an enzyme, is crucial to create strong intermolecular bonds between the free amino and polymers or oligomers resulting from the aldol condensation of glutaraldehyde. Moreover, the addition of BSA, which forms co-aggregates with an enzyme containing few lysine residues, can form CLEAs with high activity and improved mechanical properties (Hakimi et al., 2019) Also, the resistance to extreme pHs presented by CLEAs was a result of the newly formed secondary interactions between glutaraldehyde and some catalytic relevant amino acids of the enzyme (Hu et al., 2021). Although the synthesis of CLEAs is an efficient method for enzyme immobilization, there are a few limitations, such as low mechanical stability if the crosslinking was not appropriate, resulting in the leaching of the enzyme. Also, CLEAs need to be separated for reutilization from the reaction mixture *via* centrifugation or filtration, which increases the size of CLEAs due to aggregation and leads to internal mass transfer limitations. However, alternative solutions have been proposed to obtain a biocatalyst with favorable mechanical properties, where a novel strategy of magnetic crosslinked enzyme aggregates (magnetic CLEAs) was developed (Talekar et al., 2012). If the second monomer is involved in the reaction of crosslinking, such as magnetic nanoparticles, which are previously functionalized with active amino groups, together with an enzyme, they crosslink together into a three-dimensional structure, named magnetic CLEAs (mCLEAs) (Sheldon, 2011). In this regard, a further immobilization of CLEAs on solid supports of magnetic nanoparticles (MNPs) can be easily controlled and separated from the reaction mixture by applying a magnetic field, which excludes the filtration and centrifugation and thus simplifies the separation and the recovery process of mCLEAs (Han et al., 2021). The high specific surface of MNPs favors the binding efficiency of the enzyme, and the superparamagnetic behavior of the support permits the easy and selective recovery of the biocatalyst using a magnet and its subsequent reuse in more catalytic cycles (Primožič et al., 2020). Typically, the preparation procedure of enzyme immobilization in the form of CLEAs and further with the addition of MNPs to mCLEAs were performed according to the scheme shown in Figure 1.

Generally, the properties of an immobilized enzyme may be different from those of the same enzyme in free solution and depend both on the method of immobilization and the nature of the enzyme. A reduction in specific activity may occur as an enzyme is immobilized, mainly if a chemical process is involved since the conditions might cause some denaturation (Palmer and Bonner, 2007).

There exist enzymes with high importance in different application fields. Enzymes used in this research are classified into three different functional classes of enzymes, where laccase belongs to a group of oxidoreductases and is involved in the oxidation of various phenolic compounds. In this context, β-gal and cellulase are hydrolases that catalyze the hydrolysis of various bonds and TGM is an enzyme in the class of transferases that catalyze the formation of an amide bond between the y-carboxamide groups of peptide-bound glutamine residues (Brubaker and Messersmith, 2012). Briefly, TGMs are Ca^2+^-dependent enzymes that post-translationally modify specific glutaminyl (Gln) side chains in proteins by deamidation, transamidation, or esterification (Lorand and Graham, 2003). Afterwards, β-gal (EC 3.2.1.23), also known as lactase, is classified as part of the glycoside hydrolase family due to its high specificity towards galactosides with two classical catalysis functions: hydrolysis of lactose into glucose and galactose and transgalactosylation, which involves cleaving of galactosyl and subsequently transferring it onto a lactose molecule, thus forming *trans*-galactooligosaccharide (GOS). Due to the dual use of β-gal, this enzyme is important in the food (dairy) industry (Xu et al., 2020). Moreover, it is broadly used in the production of lactose-hydrolyzed products for lactose-intolerant people or lactase-deficient people. Furthermore, use of scCO_2_ improves mass transfer due to higher diffusivity resulting in shorter processing time with consequent preservation of essential food elements, which means minimal degradation of nutritional and organoleptic properties. Therefore, scCO_2_ technology is also suitable for β-gal hydrolysis involved in dairy processing as it allows microbial and enzymatic inactivation (Amaral et al., 2017). The use of immobilized β-gal for hydrolyzing milk lactose is usually targeted to reduce enzyme and processing costs (Harju et al., 2012). Based on these premises, β-gal can be immobilized on an extremely wide variety of supports employing all the primary immobilization techniques such as adsorption, entrapment, and covalent linkage. Nonetheless, very few can be considered as suitable for commercial use because the physical properties of the supports themselves are not suitable for industrial processes, e.g., poor mechanical strength (Mahoney, 1997). In this regard, β-gal CLEAs would be potentially suitable for the hydrolysis process, as it does not include a typical carrier as other types of immobilizations.

In addition, cellulases have an essential role in maintaining the carbon balance in nature and are produced mainly by fungi and bacteria. It has been widely accepted that three types of enzymes, namely, endoglucanases (EC 3.2.1.4), exoglucanases (EC 3.2.1.91), and β-glucosidases (EC 3.2.1.21), act synergistically to convert cellulose into β-glucose. In spite of that, cellulases have an enormous biotechnological potential for various industries, including chemicals, fuel, food, brewing and wine, animal feed, textile and laundry, pulp, and paper, as well as in the agriculture sector (Hojnik Podrepšek et al., 2019). Laccase (EC 1.10.3.2) belongs to the class of multicopper lignin-modifying enzymes catalyzing the oxidation of phenol-like compounds, aromatic amines, and some inorganics. Over the last decades, the use of laccase has been explored for the biodegradation of xenobiotics, for bleaching in the pulp and paper industry, and for decolorization in the textile industry (Cabana et al., 2007). In general, enzyme immobilization generally results in enzyme stabilization against thermal and chemical denaturation and in kinetic behavior modifications.

Therefore, in this paper, we combined many years of our experience and results based on the immobilization of enzymes into CLEAs and compared the process of immobilization of different enzymes, such as cellulase, β-gal, TGM, and laccase. In addition, storage stability testing of CLEAs and mCLEAs is also provided. One of them is exposure to scCO_2_, which has proven appropriate in the past as it offers environmental advantages over chemical solvents and provides enhanced separation and chemical selectivity. Furthermore, the combination of scCO_2_ and enzymes offers significant potential for developing environmentally responsible bio-processing (Baig et al., 2011). Moreover, the stability of biocatalysts is one of the crucial criteria for their possible application in industrial enzyme-catalyzed processes (Hobbs et al., 2006). Therefore, the results of residual activity of CLEAs and mCLEAs synthesized from different enzymes and exposed under various exposure conditions in a non-aqueous medium, such as scCO_2_, are presented in this study. To the best of our knowledge, such a comparative study has not been published so far. During the research, it was found that obtained results in the synthesis of TGM CLEAs and TGM mCLEAs are unique and could not yet be traced in any publication. It also describes the difference between synthesized CLEAs and the use of different enzymes, which gives us a new insight that each enzyme responds differently according to immobilization. Additionally, the differences in the optimization of immobilization procedures of each enzyme were explained, and storage stability and stability with repeated usage of immobilized enzyme were evaluated, which is crucial for further use in different applications.

## 2 Materials and Methods

### 2.1 Materials

Cellulase (Cellusoft conc. L) (EC 3.2.1.4) was kindly donated by Novozymes A/S (Denmark). TGM (EC 2.3.2.13) and Probind TXo were obtained from BDF Natural Ingredients (Spain). Enzyme β-gal (EC 3.2.1.23) from *Aspergillus oryzae* ≥ 8.0 units/mg solid and laccase (EC 1.10.3.2) from *Trametes versicolor* were purchased from Sigma-Aldrich (Germany). Egg albumin and pentaethylenehexamine (PEHA) were obtained from Acros Organics (Germany). Moreover, ethanol (99.8%) was purchased from Kefo (Slovenia); hydrogen peroxide solution (30%), methanol, acetone, and 2,2′-Azino-bis(3-ethylbenzothiazoline-6-sulfonic acid) diammonium salt (ABTS) were purchased from Merck Chemical Company (Germany). 2-Propanol was obtained from Kemika (Croatia), and carbon dioxide 2.5 (purity 99.5%) was supplied by Messer MG (Ruše, Slovenia). Other reagents, including glutaraldehyde GA (25%), bovine serum albumin (BSA), ferrous chloride tetrahydrate (FeCl_2_·4H_2_O), ferric chloride hexahydrate (FeCl_3_·6H_2_O), ammonia solution [25% (w/w)], sodium silicate (Na_2_SiO_3_), amino silane coupling agent [3-(2-aminoethylamino)-propyl-dimethoxymethylsilane or AEAPS)], sodium cyanoborohydride solution (NaBH_3_CN), ortho-nitrophenyl-β-galactoside (ONPG), Tris buffer, CBZ-glutaminylglycine (CBZ-Gln-Gly), hydroxylamine hydrochloride, glutathione, calcium chloride dihydrate, l-glutamic acid g-monohydroxamate, and trichloroacetic acid, were purchased from Sigma-Aldrich (Germany). All solutions were freshly prepared every day with Milli-Q water. All other reagents were of analytical grade and were obtained from either Sigma-Aldrich or Acros Organics.

### 2.2 Methods

#### 2.2.1 CLEAs Preparation

In general, the preparation of CLEAs involves two steps: (1) physical aggregation/precipitation of the enzyme and (2) crosslinking. In addition, all reactions were performed at room temperature (T = 25°C) using pre-chilled solvents. Enzyme precipitation was conducted using 90% (v/v) of precipitation reagent and 10% (v/v) of the enzyme. The tested precipitants were ethanol, acetone, methanol, 2-propanol, propanol, tetrahydrofuran, and ammonium sulfate. The residual activity of the precipitated enzyme was calculated between the activity of the resuspended precipitated enzyme (*U*) and the activity of the free enzyme (*U*_*0*_), which was used before the precipitation process, as given in Eq. 1:

$$Residual\;activity\;of\;precipitated\;enzyme\;\left(\%\right)=\frac{Activity\;of\;precipitated\;enzyme\;\left(\text{U}\right)}{Activity\;of\;free\;enzyme\left(U_{0}\right)}*100$$ (1)

**FIGURE 2:**
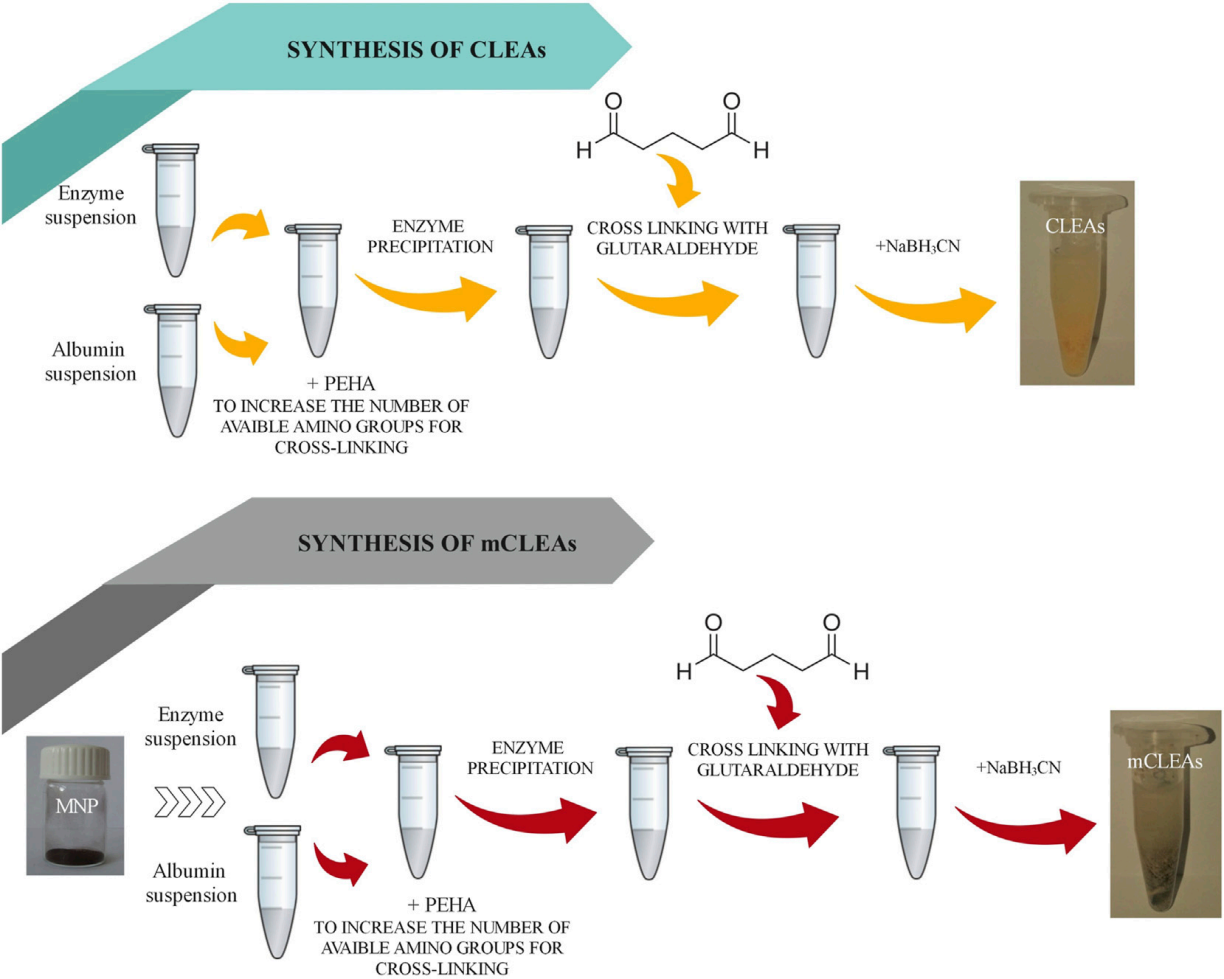
Schematic representation of CLEAs and mCLEAs preparation.

GA was used as a crosslinking reagent, where the crosslinking reaction was performed in the presence of PEHA and albumin, which is demonstrated in Figure 2. Cold NaBH_3_CN solution was used as the reducing agent. Finally, the CLEAs were recovered by centrifugation, dispersed in 0.2 M phosphate buffer solution (PBS), and stored at 4°C for subsequent activity analysis.

#### 2.2.2 Magnetic CLEAs Preparation

Maghemite nanoparticles were synthesized by hydrothermal coprecipitation of Fe^2+^ and Fe^3+^ ions in the presence of ammonium. At first, 2.684 g of FeCl_2_*4H_2_O and 3.11 g of FeCl_3_*6H_2_O were dissolved in double-deionized Milli-Q water and thoroughly mixed at room temperature. The initial pH value was adjusted to 3 (±0.1) with stirring. Then, the pH was increased rapidly to 11 (±0.1) by adding 25% of the ammonia solution directly into the solution of iron ions under vigorous stirring. Finally, the γ-Fe_2_O_3_ precipitates were washed with deionized water, collected by magnetic decantation, and dried in a vacuum oven at 70°C (Hojnik Podrepšek et al., 2020). Afterwards, maghemite nanoparticles obtained previously were dispersed in Milli-Q water and then coated with citric acid (γ = 0.5 g ml^−1^) that served as a surfactant, respectively. Later, after surfactant coating, the maghemite nanoparticles were centrifuged at 3,000 rpm for 5 min, and the precipitates at the bottom of a centrifuge tube were removed, whereas the magnetic fluid composed of stable maghemite nanoparticles was utilized in the next step of surface functionalization with silica. Therefore, in order to obtain a uniformly distributed functional layer of silica onto the surface of maghemite nanoparticles, sodium silicate and organosilane AEAPS were consecutively added into magnetic suspension. The reaction was allowed to proceed at 90°C under continuous mechanical stirring, respectively. In the end, the suspension was cooled down, and hydrochloric acid was added drop-wise to adjust the pH value to 7. Finally, the resulting amino functionalized maghemite nanoparticles were separated by a permanent magnet (2,000 Oe) and dried at air (Šulek et al., 2010). Thus, silanation reaction by amino silane coupling agent took place in order to provide highly functionalized γ-Fe_2_O_3_ nanoparticles ready for use in the synthesis of mCLEAs.

Furthermore, amino functionalized MNPs (5 mg) and enzyme in phosphate buffer were mixed. Next, an appropriate concentration of GA was added for crosslinking, followed by shaking at room temperature for a proper time. Afterwards, the mCLEAs synthesis process was continued according to the same procedure as for CLEAs synthesis. The scheme from Figure 2 demonstrates the synthesis steps of CLEAs and mCLEAs and the comparison in the synthesis process.

The resulting biocatalyst combines the relevant catalytic properties of CLEAs (as excellent stability and feasibility for their reutilization) and the magnetic character. Thus, the final product (mCLEAs) is more stable than the free enzyme, easily recoverable from the reaction medium, simply by using a magnet and reusable for new catalytic cycles.

### 2.3 Immobilization Efficiency

In order to improve the immobilization efficiency, reaction conditions for precipitants were needed to be optimized. Thus, the optimization was carried out by changing the concentration of the enzyme and the ratio between solvent reagents and enzyme preparation. Specifically, protein concentrations were determined by the spectrophotometric method of Bradford (1976). Measurements were carried out using a UV-Vis spectrophotometer (Varian Cary Probe 50, Agilent technologies) at a wavelength of 595 nm, where BSA was used as a standard in the concentration range of 0.0–1.0 mg ml^−1^ to construct the calibration curve. The resulting protein concentrations in obtained supernatant represent the concentration of the proteins, which are not crosslinked as CLEAs and mCLEAs. The immobilization efficiency was calculated as the ratio between the initial value of the enzyme concentration that was used for the synthesis of CLEAs, mCLEAs, and values in supernatants after the synthesis and was calculated by Eq. 2:

$$IE\left[\%\right]=\frac{Total\;protein\;concentration\;of\;immobilized\;enzyme}{Total\;protein\;concentration\;of\;free\;enzyme}*100$$ (2)

All protein determination experiments were performed in triplicate. Data were expressed as the means ± standard deviations of three replicates and varied for less than ±3%.

### 2.4 Enzyme Activity

The activities of free enzyme, CLEAs, and mCLEAs were determined through spectrophotometric activity assays on a UV-Vis spectrophotometer (Varian Cary Probe 50, Agilent technologies) at different wavelengths.

#### 2.4.1 Cellulase Activity

The activities of free cellulase (dissolved in PBS buffer, pH = 7) and the immobilized cellulase in CLEAs and mCLEAs were determined by cellulase activity assay, using cellulose as a substrate (Hojnik Podrepšek et al., 2019). The detailed assay procedure is as follows: 1 ml of enzyme sample suspended in PBS buffer was added into 4 ml of 5% Sigmacell cellulose solution (dissolved in 50 mM sodium acetate buffer, pH = 5). The temperature of the solution was maintained at 37°C for 2 h. Then, the mixtures were centrifuged to clarify the supernatant. The activities of free and immobilized enzymes were determined using a UV-spectrophotometer at 340 nm. Enzymatic activity unit (U) is defined as the amount of enzyme cellulase to liberate 1.0 µmol of glucose from cellulose per hour at pH 5.0 at 37°C (2-h incubation time).

Activity (U/ml) of cellulase was calculated according to the Eq. 3,

$$Cellulase\;activity\left(\frac{U}{mL}\right)=\frac{\left(A_{s}\left(340nm\right)-\;A_{b}\left(340nm\right)\right)\;0.775*5*df}{6.22*2*1*0.025}$$ (3)

where 0.775 was a final volume of glucose assay reagent and supernatant in milliliters, five was the total volume of the reaction mixture, *df* was dilution factor, 6.22 was a millimolar extinction coefficient of β-NADH at 340 nm, two was the conversion factor, one was the volume of enzyme suspension, and 0.025 was the volume in a milliliter of supernatant.

#### 2.4.2 β-gal Activity

The activity of β-gal was determined based on ortho-nitrophenyl-β-galactoside (ONPG) as a colorimetric and spectrophotometric substrate in a specific reaction mixture for the enzyme β-gal. The reaction mixture consisted of 2.0 mM ONPG dissolved with 0.01% (w/v) BSA in 0.1 M sodium acetate buffer and 200 μl of enzyme solution and was stirred for 10 min. After stirring, 4 ml of sodium carbonate (1 M) was added, and the β-gal activity was determined at 37°C and pH = 6.0. The absorbance was measured with a UV-spectrophotometer at a wavelength of 405 nm (Križnik et al., 2018).

Activity (U/ml) of the β-gal was calculated according to Eq. 4,

$$\beta -galactosidase\;activity\;\left(\frac{U}{mL}\right)=\frac{A_{s}{\left(405nm\right)}_{-}A_{b}\left(405nm\right)*5*df}{10*4.6*0.2}$$ (4)

where five was the total volume of the sample (ml), 10 was stirring time (min), 4.6 was a millimolar extinction coefficient of ortho-nitrophenol absorbance at 405 nm, *df* was dilution factor, 0.2 was the volume of the enzyme (ml), *A*_*s*_ was the absorbance of the sample, and *A*_*b*_ was the absorbance of a blank.

#### 2.4.3 TGM Activity

The TGM activity was determined by a colorimetric hydroxamate procedure in which Z-Gln-Gly was used as the amine acceptor substrate and hydroxylamine was used as an amine donor. In the presence of TGM, hydroxylamine was incorporated into Z-Gln-Gly to form Z-glutamyl-hydroxamate-glycine, which developed a colored complex with iron (III) detectable at 525 nm on UV-spectrophotometer. One unit of TGM activity was defined as the amount of enzyme that causes the formation of 1.0 μmol of hydroxamate per minute by catalyzing the reaction between Z-Gln-Gly and hydroxylamine at 37°C and pH 6.0. l-glutamic acid γ-monohydroxamate was used as a standard probe (Gajšek et al., 2019).

Activity (U/ml) of the TGM was calculated according to Eq. 5,

$$Transglutaminase\;activity\;\left(\frac{U}{mL}\right)=\frac{A_{test}\left(525nm\right)*df}{A_{std}\left(525nm\right)*1.1*10*1.23}$$ (5)

where *A*_*test*_ (525 nm) was the absorbance of the sample measured at 525 nm, *df* was dilution factor, *A*_*std*_ (525 nm) was the absorbance of the standard, 1.1 was the volume of standard (ml), 1.23 was the volume of the color mix (ml), and 10 was reaction time (min).

#### 2.4.4 Laccase Activity

The laccase activity was spectrophotometrically determined by monitoring the oxidation of 2,2′-azino-bis(3-ethylbenzothiazoline-6-sulfonic acid) diammonium salt (ABTS). The reaction mixture contained 1 mM ABTS, 0.1 M sodium acetate buffer, and an enzyme sample. Before the measurement, 200 μl of ABTS was added, the content was stirred for 1 min using a vortex, and the absorbance of the sample was measured at 420 nm on a UV-spectrophotometer (Primozic et al., 2019). One unit of laccase activity is defined as the amount of enzyme oxidizing 1 μmol of ABTS per minute under standard assay conditions.

Activity (U/mg_enzyme_) of the laccase was calculated according to Eq. 6,

$$Laccase\;activity\;\left(\frac{U}{mg_{enzyme}}\right)=\frac{\left(\frac{A_{s}}{t}\right)*1.2*df}{3.6*m_{enyzme}}$$ (6)

where *A*_*s*_ was the absorbance of sample, *t* was reaction time (min), 1.2 was total volume of reaction mixture (ml), *df* was dilution factor, 3.6 was an extinction coefficient, and *m*_*enzyme*_ was the mass of enzyme in the reaction mixture (mg).

In the results, the average values of the residual activity are given and may vary for ±3% due to the absolute mistake in the execution of the experiment.

### 2.5 Characterization

Scanning electron microscopic (SEM) images of TGM CLEAs and TGM mCLEAs were recorded using a SIRION, 400 NC, FEI microscope. Fourier-transform infrared (FT-IR) spectra were detected over a range of 4,000–400 cm^−1^ on a FT-IR spectrophotometer Perkin Elmer 1,600 using ATR accessory for liquid analysis. The thermogravimetric analysis (TGA) was performed on a Mettler Toledo TGA/DSC one thermogravimetric analyzer under N_2_ atmosphere and a heating rate of 10°C/min.

### 2.6 Stability Determination of CLEAs and mCLEAs in scCO_2_

The stability of immobilized enzyme preparations was determined using standard procedure. The test tubes containing CLEAs and mCLEAs were placed in the high-pressure batch reactor at different pre-set temperatures for specified exposure time (Supplementary Figure S1). The high-pressure reactor was temperature- and pressure-regulated with a volume of 60 ml.

After exposure at a certain treatment time in scCO_2_, a slow CO_2_ expansion was carried out, samples were taken out from the reactor, and the activity of β-gal, TGM, and laccase CLEAs and mCLEAs was assayed based on specific enzyme activity assays. In addition, the activity of the exposed immobilized enzyme was compared to the activity of the immobilized enzyme before exposure to scCO_2_ and was defined as residual activity (%). The percent of residual activity in CLEAs and mCLEAs was calculated, as given in Eq. 7:

$$Residual\;activity\;\left(\%\right)=\frac{total\;activity\;of\;CLEAs/mCLEAs\;after\;scCO_{2\;}treatment\;\left(\text{U}\right)}{total\;activity\;of\;CLEAs/mCLEAs\;before\;scCO_{2}treatment\left(U_{0}\right)}*100$$ (7)

All experiments were done in triplicate, and the error bar represents the percentage error (±5%) in each set of readings.

### 2.7 Determination of Storage Stability

Storage stability of CLEAs and mCLEAs after storage at 4°C was determined with freshly prepared CLEAs and mCLEAs from cellulase, β-gal, TGM, and laccase. Samples were stored in the fridge at 4°C, and then residual activity was determined through spectrophotometric activity assays for 44 days. The initial enzyme activity value of CLEAs and mCLEAs was taken as control (100%) to calculate the residual activity. All experiments were done in triplicate, and the error bar represents the percentage error (±5%) in each set of readings.

Statistical analysis was additionally performed in R version and Rstudio to evaluate the differences in residual activity between CLEAs and mCLEAs and between enzyme groups. Residual activity data distribution was evaluated with Shapiro–Wilk statistical test. Normal data distribution was observed, where data were presented with mean and standard deviation. Paired *t*-test was performed to compare residual activity between CLEAs and mCLEAs, according to sample time, coupled by week. Analysis of variance (ANOVA) and Tukey’s honest significance difference (HSD) test were performed to compare the differences between types of enzymes.

### 2.8 Repeated Usage of CLEAs and mCLEAs

The main advantage of immobilized enzyme application, besides their stabilization, is the possibility of using them for long periods. Therefore, the possibility of reusing CLEAs and mCLEAs was evaluated through a different number of reaction cycles. CLEAs and mCLEAs were prepared according to the procedure with optimal parameters. The activity of the CLEAs and mCLEAs was determined after each reaction cycle, where, after the first activity assay, CLEAs were centrifuged and mCLEAs were magnetically separated before being reused. The procedure was repeated several times in succession, and the residual activity of CLEAs and mCLEAs was determined.

Enzyme activity after each cycle was expressed as residual activity (%) and calculated between enzyme activity after the last cycle and the enzyme activity after the first cycle by taking the enzyme activity of the first cycle as 100%.

## 3 Results

### 3.1 Optimization of the CLEAs and mCLEAs Synthesis

The preparation of CLEAs is simple and combines a two-step process (enzyme precipitation and crosslinking) into a single operation. In the first step, the soluble enzymes were precipitated to form enzyme aggregates by water-miscible organic solvents or salts. Crosslinking (second step) includes the addition of a certain amount of GA (crosslinker) after the precipitation step.

#### 3.1.1 Precipitation

The most suitable precipitating reagent for CLEAs synthesis has been experimentally determined. Moreover, by using the precipitation reagent where precipitation occurred, the activity of the precipitated enzyme was determined. The tested precipitants were acetone, ammonium sulfate, 1-butanol, ethanol, methanol, 1-propanol, and 2-propanol. Briefly, each class of precipitants (salts and organic solvents) decreases protein solubility by a different mechanism. Using salt as precipitation reagent, kosmotropic ions bind water more tightly than water binds itself and the surface tension of the solution increases, effectively competing with the protein surface for hydration of water molecules. As less water becomes available to hydrate the protein surface, the protein molecules self-associate and precipitate. When organic solvents, such as alcohols, have been used for protein precipitation, the dielectric constant of the solution decreases. As the dielectric constant decreases, the solution becomes a poorer solvent for the protein. Consequently, the relative favorability of protein–protein interactions increases and the protein precipitates (Kramer et al., 2012). The efficiency of precipitation was determined by the highest residual activity of the resuspended enzyme. Table 1 shows residual activities of resuspended enzymes after precipitation in connection with different precipitation reagents. Typically, precipitation is affected by changes in the pH, ionic strength, temperature of the solvent, protein concentrations, and precipitant. Certainly, the enzyme behavior in precipitation often depends more than just on thermodynamics. Factors such as the input concentration and rate of precipitate addition, the contact mechanism, the duration and level of mixing, and the final recovery of precipitate are important (Niederauer and Glatz, 1992).

**TABLE 1:** The effect of different precipitation reagents on enzyme precipitation performance.

Precipitation reagent/Enzyme	Cellulase	β-galactosidase	Transglutaminase	Laccase
		Residual activity of precipitated enzyme (%)		
Acetone	83	101	168	20
Ammonium sulfate	79	—	—	—
1-buthanol	—	—	—	56
Ethanol	84	103	173	68
Methanol	—	—	179	32
1-propanol	84	102	178	65
2-propanol	83	102	231	63

Enzyme precipitation was being estimated to find the precipitation reagent that could yield more stable CLEAs from the specific enzyme.

The lowest residual activities were expressed by the precipitated laccase and were between 20% and 68%. Therefore, it can be stated that the precipitation depends on the type of enzyme and that parameter optimization is required in the synthesis of CLEAs for each enzyme separately. Sulaiman and co-workers reported that the activity for precipitated cellulase using ammonium sulfate retained 60.1% of the original activity (Sulaiman et al., 2014). For instance, in our study, residual activity of precipitated cellulase was higher (79%). In addition, precipitation with ammonium sulfate has no adverse effects on enzyme activity and does not denature protein structures. The protein functionality is fully recovered upon resolubilization in appropriate physiological buffers (Righetti and Boschetti, 2013). Typically, TGM catalyzed transamidation between glutamine and lysine residues that can lead to the formation of covalent side-chain bridges between protein units; in this sense, TGM’s function as nature’s catalysts is to glue proteins together, and therefore to generate crosslinked supramolecular protein assemblies (Lorand and Graham, 2003). For this reason, high values of residual activity after precipitation are predicted. From the results in Table 1, it can be stated that the residual activity of precipitated TGM is much higher using various precipitants than in the precipitation of other enzymes and reached up to 231% of the original activity, and the rest were above 160%. Next, β-gal showed residual activities slightly above 100% in all precipitation reagents, as well. On this basis, we assumed that one could obtain increased activity of the synthesized CLEAs for these two enzymes. The results indicated that 2-propanol and ethanol are the most suitable precipitating reagents for the precipitation of the enzymes cellulase, β-gal, TGM, and laccase, since a high residual activity of precipitated enzymes was obtained. These data clearly demonstrate that the dielectric constant of a solvent is an important physicochemical parameter. Dielectric constants of 2-propanol (ε = 18) and ethanol (ε = 25) increased the electrostatic forces of attraction between isoelectric protein molecules and thus advances a mechanism for the precipitation of proteins (van Oss, 1989; Mohsen-Nia et al., 2010). Moreover, 2-propanol is often found to be the optimal precipitant for immobilization applications (Rathankumar et al., 2019), and it is considered as a cheaper precipitating reagent compared to others. Based on the highest activities of the precipitated enzyme obtained, the precipitating reagent that is most preferential for the precipitation of the enzyme was determined. Results of this work have shown that ethanol is the most suitable for cellulase precipitation, 1-propanol is the most suitable for precipitation of β-gal and laccase, and 2-propanol is the most suitable precipitating reagent for precipitation of TGM. Therefore, selected precipitants increased enzyme activity and were also further used for specific enzymes in the crosslinking process.

In the synthesis of CLEAs, precipitation is followed by crosslinking with a crosslinking reagent, which is usually GA. Therefore, the optimization of the crosslinking process was conducted in the next phase to obtain CLEAs of different enzymes with improved activity and stability characteristics.

#### 3.1.2 Crosslinking

One of the most effective protein crosslinking reagent is GA, a universal bifunctional crosslinker with high reactivity. It reacts rapidly with amine groups at around neutral pH and is more efficient than other aldehydes in generating thermally and chemically stable crosslinks (Migneault et al., 2004). GA reacts primarily with amino groups of proteins in biological systems (Cheung and Nimni, 2009). Because the molecular weight of GA used as a crosslinking reagent is meager than the enzyme’s molecular weight, it is assumed that only the active enzyme is crosslinked in the form of CLEAs (Sheldon et al., 2006). However, a very high concentration of GA can cause irreversible enzyme deactivation. Therefore, the effective concentration of GA is essential to provide biomechanical stability (Lee et al., 2017). Crosslinking is incomplete when a very low concentration of GA is used. Thus, optimization of GA concentration is necessary in order to avoid a decrease in the relative activity of CLEAs or enzyme inactivation.

The crosslinking reaction of cellulase was performed for 3 h at room temperature. The effect of GA concentration as a crosslinking reagent on the immobilization efficiency and activity of cellulase CLEAs was studied within the GA concentrations of 0.625%–20% (v/v) in the presence of ethanol, methanol, and propanol as precipitating reagents. The relative activity of CLEAs increased to 94% when a GA concentration of 0.625% (v/v) with ethanol as precipitating reagent was used (Table 2). Upon further increase of GA concentration, enzyme inactivation was observed as relative activity began to decline. Using methanol as precipitating reagent, the relative activity of CLEAs increased with a GA concentration of 3.75% (v/v), with the highest value of 62%; using propanol as precipitation reagent, the highest activity of CLEAs (84%) was obtained at a GA concentration of 1.25% (v/v). The study performed by Perzon and co-workers demonstrated the influence of process parameters as different precipitants on the performance of the CLEAs cellulase. In this regard, the activity of CLEAs precipitated with 50% w/v polyethylene glycol (PEG) was positively correlated with the increasing amounts of GA, while the crosslinking time had no effect. The CLEAs from 60% w/v tert-butyl alcohol precipitates were inactive. The highest activity recovery from PEG- and 40% w/v ammonium sulfate-based CLEAs was 17% and 29% relative to the free enzyme, respectively (Perzon et al., 2017). In addition, Shuddhodana and co-workers demonstrated the preparation of CLEAs of commercial cellulase mix, where the activities of β-glucosidase, endoglucanase, and xylanase were determined by performing assays at varying substrate concentrations. Various concentrations of BSA (25–125 mg/ml) and GA (0.25%–1.25% w/v) and incubation time (2–6 h) were selected as design parameters to prepare CLEAs. It was found that ammonium sulfate (90% saturation) was the precipitant of choice with 95% activity recovery in the precipitate and 33% activity recovery in the form of CLEAs (Shuddhodana et al., 2018). In contrast, the obtained results of the relative activity of CLEAs cellulase in our study are much higher compared to the results previously described.

**TABLE 2:** Optimal condition parameters for CLEAs and mCLEAs synthesis.

Optimum parameters	CLEAs cellulase	mCLEAs cellulase	CLEAs β-gal	mCLEAs β-gal	CLEAs TGM	mCLEAs TGM	CLEAs laccase	mCLEAs laccase
Enzyme concentration (mg/ml)	42	42	50	50	200	200	20	20
Precipitation reagent	Ethanol	Ethanol	1-propanol	1-propanol	2-propanol	2-propanol	1-propanol	1-propanol
Concentration of GA (%)	0.625	0.7	1.5	1.5	2.0	10.0	10.0	10.0
Crosslinking time (h)	3	3	2	2	3	3	3	3
Immobilization efficiency (%)	78	94	98	99	95	90	89	69
Relative activity (%)	94	37	118	103	63	73	46	30

The immobilization efficiency during the crosslinking optimization process of laccase was 78% with ethanol and 89% with methanol, respectively. The maximum activity of CLEAs was 62% when using ethanol [2% (v/v) GA] as a precipitating reagent. However, the relative activity of laccase CLEAs, using the precipitating reagent 1-propanol [2% (v/v) GA] was 26%. Thus, relative activities with precipitation reagents ethanol and 1-propanol [5% (v/v) GA] were 41% and 46%, while the relative activities of CLEAs with the use of ethanol [10% (v/v) GA] and 1-propanol [10% (v/v) GA] as precipitation reagents reached 28% and 44%, respectively. Furthermore, the yield of CLEAs can be improved in two ways, either by increasing the concentration of crosslinker or by increasing the lysine residue on the enzyme surface. To form irreversible crosslinking, sodium cyanoborohydride (NaBH_3_CN) was used to reduce Schiff’s base (Husain and Ullah, 2019). During crosslinking of laccase with the enzyme concentration of 20 mg/ml, 1-propanol was used as a precipitation reagent with 5% (v/v) GA. The concentration of 10% (v/v) NaBH_3_CN and crosslinking time of 3 h showed optimal reaction parameters for the synthesis of CLEAs and resulted in the highest relative activity of 46%. Reducing the volume fraction of NaBH_3_CN to 5% (v/v) compared to the addition of 10% (v/v) NaBH_3_CN negatively affected the relative activity of the CLEAs. These results differ from those obtained by Cabana and co-workers, who observed the 60% activity recovery of laccase CLEAs, precipitated with PEG with a GA of 200 µM. Moreover, their study demonstrated that the addition of BSA improved the stability of the immobilized laccase. For example, the stability of the CLEAs supplemented with BSA at 1 mg U^−1^ was 50% higher than that of the CLEAs without BSA (Cabana et al., 2007). The laccase used in their study was from *Coriolopsis polyzona* origin, which differs from the laccase used in the present study. In this regard, it is important to emphasize that the source of laccase is crucial for the activity of synthesized CLEAs.

In the optimization of TGM crosslinking with a concentration of GA between 1.027% (v/v) and 2.015% (v/v), a higher concentration of GA resulted in increased activity of the immobilized enzyme. Based on the results, the optimal concentration 2.015% (v/v) of GA was determined, with the highest CLEAs activity achieved using ethanol as precipitating reagent. Furthermore, it was observed that by increasing the GA concentration to 2.28% (v/v), the activity of the crosslinked enzyme decreased. If the optimal GA concentration was exceeded, there was a loss of flexibility of the crosslinked molecules, which subsequently led to a loss of CLEAs activity. Therefore, a 2.015% (v/v) of GA concentration was subsequently used, and NaBH_3_CN concentration was continued to be optimized. In addition, the effect of different concentrations of 0.1 M NaBH_3_CN [0.07%, 0.11%, 0.14% and 0.17% (v/v)] was tested. The highest relative activity, 53% of TGM CLEAs in 2-propanol, was obtained by the addition of 0.14% (v/v) of NaBH_3_CN, with 98% immobilization efficiency. After the addition of NaBH_3_CN concentration of 0.17% (v/v), an increase in the activity with the precipitating reagent 2-propanol to 63% was observed (Table 2). The immobilization efficiency under these conditions achieved 95%. To the best of our knowledge, no publication describing the optimization of reaction parameters for the preparation of TGM CLEAs has been published so far. Therefore, our results are unique and cannot be compared with other authors.

In the optimization of β-gal CLEAs, first, the concentration of enzyme was optimized. It was found that the highest activity (103%) was achieved when acetone was used as a precipitating reagent and slightly lower activity (100%) was obtained when 1-propanol was used as precipitation reagent. The activity of CLEAs was 10%–12% higher with the use of 50 mg/ml of β-gal, compared to a higher β-gal concentration of 100 mg/ml. Moreover, the highest activity was achieved with 3-h crosslinking with the use of 1-propanol (103%) and the lowest was obtained with the use of 2-propanol (98%). In addition, crosslinking was also performed at a shorter time. At 2-h crosslinking, the highest activity was detected with the use of 1-propanol, which was 103%, and the lowest activity was detected with the use of 2-propanol as a precipitating reagent (98%). The immobilization efficiencies were above 99%. Crosslinking is characterized by achieving a better “tight” structure of the network at lower temperatures and higher crosslinking densities (Sandolo et al., 2009). Therefore, the crosslinking was performed at 10°C, where it was noticed that the activity of β-gal CLEAs was slightly higher. Furthermore, with the use of ethanol as precipitating reagent, the activity after 2 h of crosslinking at room temperature was 102% and 109% at 10°C. The results demonstrated that using acetone as a precipitating reagent, the activity of CLEAs at room temperature was 101% and 109% at 10°C. Also, when 1-propanol was used as precipitating reagent, the activity of β-gal CLEAs was higher (118%) at 10°C, while the activity of β-gal CLEAs was 103% at room temperature. Additionally, with the use of 2-propanol as precipitating reagent, the activity of β-gal CLEAs was 99% at 10°C, while it was slightly higher at room temperature (98%). In principle, the activity of β-gal CLEAs turned out to be lower with 2-propanol, and using 1-propanol provides optimal conditions in the synthesis of β-gal CLEAs.

The above findings suggest that reaction parameters that were the most efficient in the optimization of CLEAs are represented as the optimal reaction parameters for achieving the highest activity of CLEAs and were used for the synthesis of mCLEAs. Table 2 also shows the results of selected optimal reaction parameters for further synthesis of mCLEAs from enzymes cellulase, β-gal, TGM, and laccase. In principle, the relative activities of enzymes immobilized in the form of mCLEAs are lower, except for TGM mCLEAs, which were 10% higher. A slight reduction in enzyme activity was observed when free enzyme was crosslinked with activated maghemite nanoparticles. The crosslinking of TGM generates proteolytically resistant γ-glutamyl-ε-lysine bonds, which, together with MNPs, form a stable structure, and reflect good stability and high activities of TGM mCLEAs. In contrast, in the synthesis of mCLEAs from other enzymes, it was lower compared to the relative activity of CLEAs. Lower relative activities for mCLEAs compared to CLEAs relative activities after crosslinking could be attributed to the dilution effect on the enzyme with the addition of the MNPs due to unsettled aggregation and leakage of the enzyme (Dal Magro et al., 2018; Mehde, 2019). However, even slightly lower relative activity does not challenge the possibility offered by mCLEAs by the fact that the immobilized enzyme can be easily removed from the reaction mixture and reused. On the other hand, some authors also revealed that the addition of magnetic nanoparticles had a positive effect on the recovered activity of mCLEAs using other enzymes, such as α-amylase, xylanase, β-xylosidase, acetyl xylan esterase, and phenylalanine ammonia lyase (Talekar et al., 2012; Bhattacharya and Pletschke, 2014; Cui et al., 2014). The inhibitory effect of ferrous/ferric ions reflects the complex interactions with the enzyme that cannot be simply explained by crosslinking and paves the way for further studies.

Xie and co-workers optimized the choice of precipitants, the concentration of GA, and the concentration of cellulase in preparation of cellulase CLEAs immobilized on magnetic Fe_2_O_4_-CS microspheres. A similar observation to ours was reported with a concentration of GA, as excessive GA impacts the catalytic effect, but on the other hand, a higher concentration of GA can fix the structure of enzyme aggregate stability. In their study, GA concentration below 1% improved cellulase mCLEAs activity, where the higher activity of cellulase mCLEAs reached 46%. Moreover, the temperature was another critical parameter. However, higher temperatures can lead to enzyme denaturation with chemical bond breakdown and can cause activity loss. However, in mentioned research, it was found that the optimal temperature for the preparation of cellulase mCLEAs is 30°C, where the cellulase mCLEAs activity reached 50% of its original activity (Xie et al., 2012).

### 3.2 Characterization of CLEAs and mCLEAs

**FIGURE 3:**
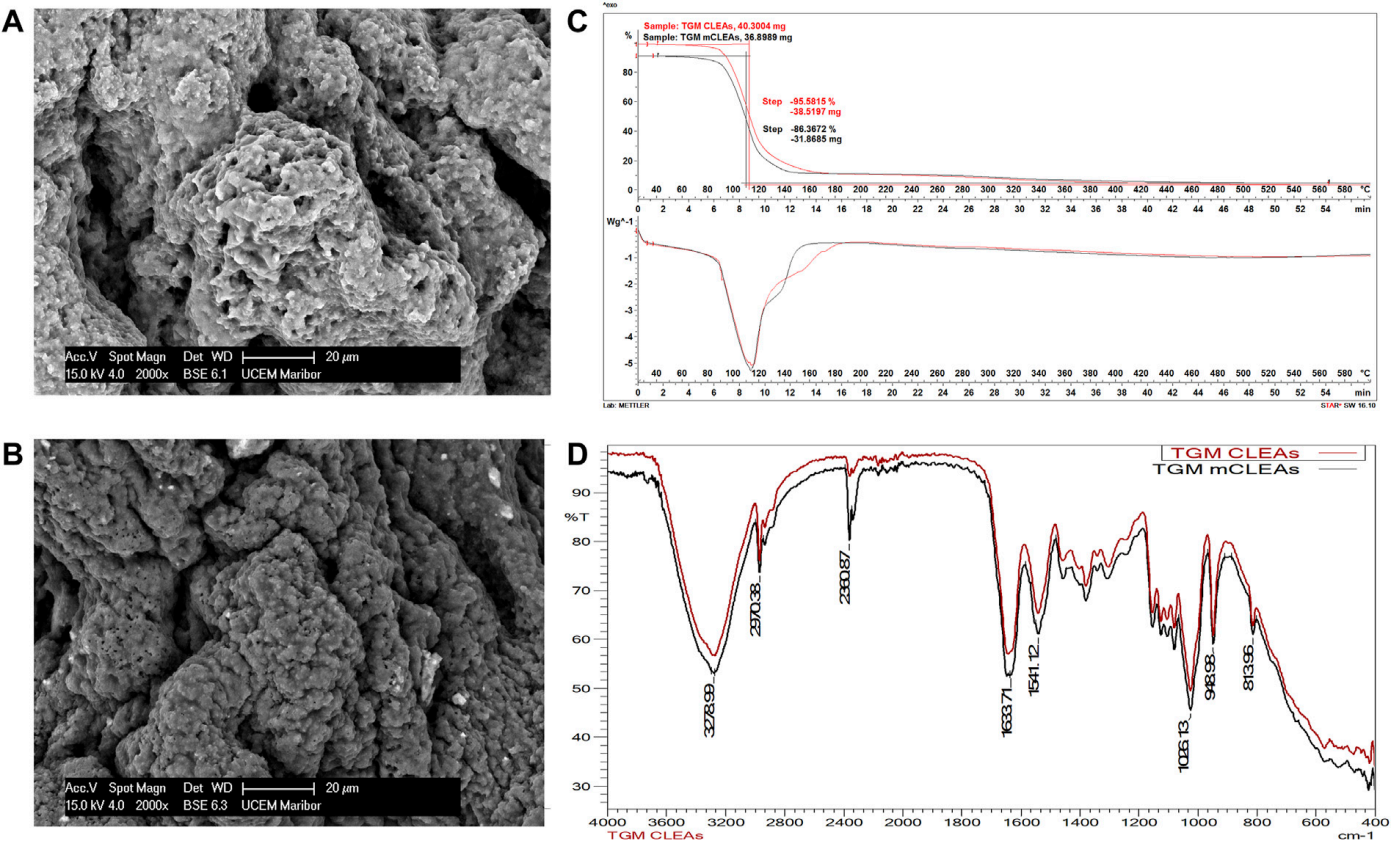
SEM images of CLEAs **(A)** and mCLEAs **(B)**, thermogravimetric and differential thermal analysis curves of CLEAs and mCLEAs **(C)**, and FT-IR spectra of CLEAs and mCLEAs **(D)**.

After synthesis of TGM CLEAs and TGM mCLEAs, characterization of the immobilized enzyme was performed to obtain the structural properties. The characteristic properties of TGM CLEAs and mCLEAs have been determined, as this has not been published in the literature so far and because it represents the highest relative activity and stability compared to the synthesized CLEAs and mCLEAs from other enzymes. The size and morphology of CLEAs and mCLEAs were investigated using SEM. As shown in Figure 3A,B, the SEM image revealed that the TGM CLEAs and TGM mCLEAs have an amorphous structure, and the mean diameter of the aggregates was approximately 40 µm. The SEM image of mCLEAs shows tiny bright parts on the periphery corresponding to MNPs. Most MNPs were crosslinked in the form of mCLEAs, which is well seen to be trapped in a crosslinked structure. At the same time, FTIR analysis (Figure 3D) also confirmed that MNPs were located inside the structure of mCLEAs, as the spectra of CLEAs and mCLEAs were very similar, with a difference in the characteristic peak indicating the presence of MNP. The absorption peaks in the FT-IR spectrum of mCLEAs at 570 cm^−1^ and 682 cm^−1^ belong to the stretching vibration of the Fe–O bond (Mehde, 2019), which indicates that the MNPs were presented in mCLEAs. In this range, the spectrum of mCLEAs differs from that of CLEAs. Additionally, all other characteristic peaks are comparable and coincide in the CLEAs and mCLEAs spectrum.

As a result of TGM crosslinking, the peak at 1,633 cm^−1^ denoted the C=O stretching mode of the carboxylic group. The peak at 2,970 cm^−1^ was assigned to the C-H stretching. Besides that, peaks that emerged at 3,478 cm^−1^ and 1,026 cm^−1^ correspond to O-H and C-O functional groups, respectively. TGA curves of CLEAs and mCLEAs in the temperature range of 30–600°C were investigated (Figure 3C). CLEAs and mCLEAs had excellent thermal stability up to 100°C. The minor weight loss below 180°C of the immobilized enzyme was due to moisture reduction, suggesting good thermal stability of CLEAs and mCLEAs for application below 180°C. Nevertheless, 95.6% of CLEAs degradation occurred overall, while mCLEAs degradation was 86.4%. The difference in thermal degradation of 9.2% was attributed to the presence of MNPs in mCLEAs, which generally show excellent thermal stability (Hu et al., 2021). The physical–chemical characterization of γ-Fe_2_O_3_ MNPs, which has been used in recent research, was performed using various analyses in our previous work (Šulek et al., 2010).

### 3.3 Stability of CLEAs and mCLEAs in scCO_2_

Shelf life and storage conditions for enzymes depend on their physical form. They are often unstable when not in their native environments, which vary considerably among enzyme type and extracellular fluids (Betancor et al., 2006). Certainly, biocatalyst stability and selectivity are the main criteria for their use in an industrial biotransformation process. The aim of this study was to explore the potential of CLEAs and mCLEAs stability in scCO_2_ for the purpose of enzymatic catalysis in scCO_2_. Therefore, the stability study was performed at 50°C, 10 and 20 MPa. After 24 h of incubation in scCO_2_ under certain conditions, the activity of the exposed immobilized enzyme in the form of CLEAs was compared to the activity of the same immobilized enzyme before exposure to scCO_2_ and was defined as residual activity (%). Subsequently, the stability of CLEAs and mCLEAs in scCO_2_ was determined for the immobilized enzyme β-gal, TGM, and laccase. The samples were exposed to scCO_2_ in the high-pressure batch reactor for 24 h, with controlled pressure release (Δp/Δt = 5 bar/min).

**FIGURE 4:**
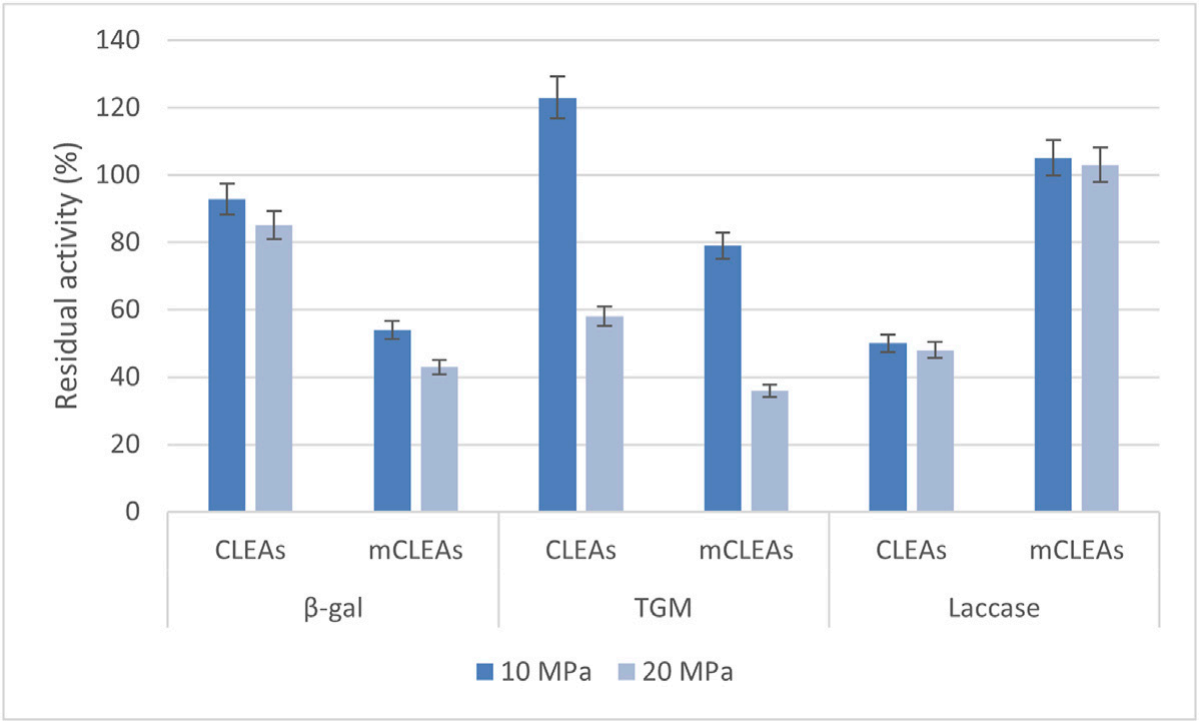
Residual activity of CLEAs and mCLEAs exposed to scCO_2_ at 50°C at different pressures.

The results in Figure 4 show the residual activity of CLEAs and mCLEAs from β-gal, TGM, and laccase exposed to scCO_2_ for 24 h at 50°C, depending on the pressure. The residual activity of β-gal CLEA after scCO_2_ treatment at 10 MPa decreased slightly to 93%. With increase in pressure to 20 MPa, the residual activity of β-gal CLEAs decreased to 85%, respectively. Some enzymes can be negatively impacted by ultrahigh pressures, which are above 40 MPa (Rezaei et al., 2007). The study performed by Xu and co-workers revealed that β-gal CLEAs precipitated with saturated ammonium sulfate under the condition of 25 mM glutaraldehyde for 3 h at 25°C obtained 23% recovered activity. Furthermore, despite the low residual activity of β-gal CLEAs, its stability was very well preserved (Xu et al., 2020).

The residual activity of β-gal mCLEAs after scCO_2_ treatment at 10 MPa decreased almost to its half, to 54%. When applying the pressure of 20 MPa, the residual activity of β-gal mCLEAs decreased even more, to 43%. Upon exposure of the immobilized enzyme in the form of TGM CLEAs to scCO_2_ at 50°C and 10 MPa, an increase to 123% in residual activity was observed. The residual activity of TGM CLEAs decreased with increasing pressure to 20 MPa, where 58% of the remaining activity was maintained. From the results of this study, it was also demonstrated that the residual activity of TGM mCLEAs decreased with increasing pressure, similarly to the exposure of TGM CLEAs. After 24 h of incubation of TGM mCLEAs in scCO_2_, the residual activity of 79% was preserved at 10 MPa and 36% at 20 MPa. The mCLEAs were less stable in scCO_2_ than CLEAs under the same conditions. Meanwhile, the residual activity of laccase CLEAs decreased to 50% of its original activity at 10 MPa, and consequently, after increasing the pressure to 20 MPa, further inactivation of laccase CLEAs occurred as it reached 48% of its original activity. Figure 4 shows excellent stability of laccase mCLEAs after 24-h incubation in scCO_2_ at 10 and 20 MPa. At 10 and 20 MPa, the residual activity of laccase mCLEAs reached 105% and 103%, slightly above the reference enzyme activity for untreated mCLEAs. The example of laccase mCLEAs shows that higher pressures used in the experiments do not turn out to be damaging for enzyme inactivation in its immobilized form. Additionally, the catalytic performance of enzyme lipase, which was treated with scCO_2_ medium at 40°C, 10 MPa for 15 min before the preparation of CLEAs, was reported by Dincyürek and co-workers. CLEAs synthesized in this way, where the enzyme was treated before the immobilization in CLEAs, prove a different approach, which consequently results in maximum lipase CLEAs activity reaching 2,279 U/g CLEAs, which is 2.2-fold compared to commercially produced CLEAs (Dinçyürek et al., 2012). On the other hand, Dijkstra presented the catalytic activity of lipase CLEA-Calb as a function of pressure, where a decrease in lipase CLEAs activities was obtained when the pressure was increased. Moreover, at a pressure above 14 MPa, almost no further decrease in activity has been observed (Dijkstra, 2006).

Obviously, the stability of the immobilized enzyme is mildly affected by the pressure. However, a slight loss of residual activity is observed for β-gal CLEAs treated in the scCO_2_, from which it can be deduced that the stability of β-gal CLEAs in scCO_2_ is adequate.

Certainly, scCO_2_ causes the inactivation of the same immobilized enzymes in the form of mCLEAs and can affect a change in the carrier structure itself, which further limits access to the active site of the enzyme. On the other hand, it allows the use of immobilized enzymes in the form of CLEAs at high pressures since more than half of the initial activity was preserved at 20 MPa. Controversially, there was even an increase in the residual activity of TGM CLEAs at 10 MPa, which indicates favorable conditions for further reactions in scCO_2_.

To date, only a few examples of the enzymatic activity of CLEAs in scCO_2_ have been reported (Chen et al., 2006; Dijkstra et al., 2007; Hojnik Podrepšek et al., 2019). The stability of immobilized laccase from *T. versicolor* in the native form and in the form of CLEAs and mCLEAs was also studied in scCO_2_ at 35°C and at 10 and 20 MPa. A study performed by Primožič and co-workers revealed that with an increase in pressure from 10 to 20 MPa, an increase in residual activity of all three laccase forms (native, CLEAs, and mCLEAs) was detected. The highest activity increase by 25% at a pressure of 20 MPa was detected for laccase mCLEAs (Primožič et al., 2020). Higher residual activity of laccase CLEAs and mCLEAs compared to our results can also be attributed to the lower temperatures of scCO_2_ exposure. According to Matsuda and co-workers, the rate acceleration of the CLEA catalyzed reaction using scCO_2_ is more prominent since the conversion of 1-arylethanols improved to 31% in scCO_2_ (Matsuda et al., 2005).

### 3.4 Storage Stability of CLEAs and mCLEAs

Storage is a crucial part of a biocatalyst because it affects the economical production costs of the enzyme. Therefore, keeping enzymes active as long as possible requires maintaining the best storage conditions. If these requirements are not satisfied, the protein can rapidly lose its ability to perform specific functions (Taheri-Kafrani et al., 2020). Proteins can lose activity as a result of proteolysis, aggregation, suboptimal buffer conditions, and inadequate storage conditions. For keeping enzyme activity intact, it is necessary to maintain active enzyme properly folded, which should prevent denaturation of CLEAs and mCLEAs. During a period of 44 days, both sample CLEAs and mCLEAs, from four different enzymes, were tested for residual activity while stored at 4°C. Tests were carried out, as described in the methods procedure, by spectrophotometric activity assays.

**FIGURE 5:**
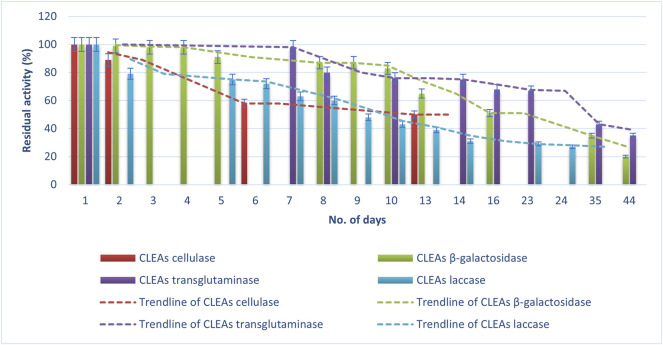
Storage stability of CLEAs at 4°C.

**FIGURE 6:**
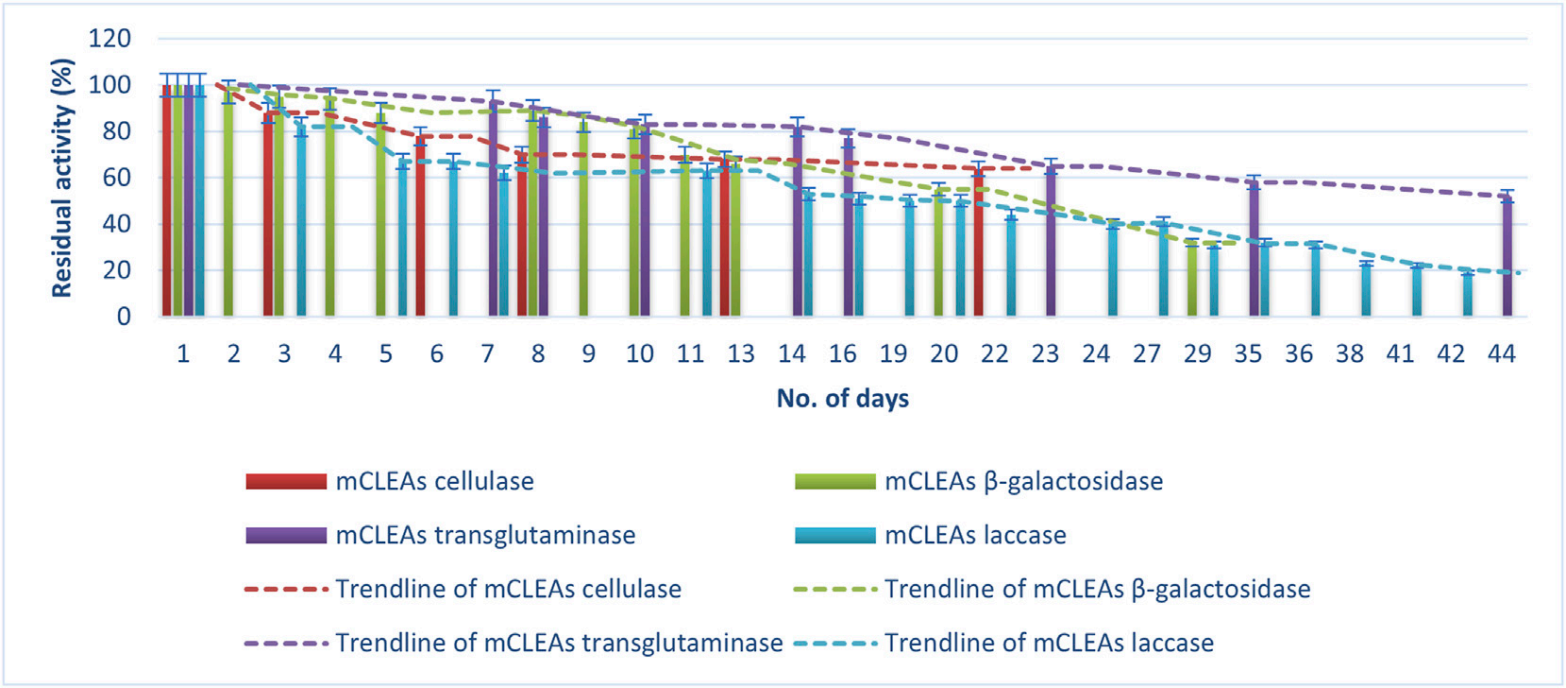
Storage stability of mCLEAs at 4°C.

As is shown in Figure 5, 6, an expected loss of activity occurs for both immobilized enzymes (CLEAs and mCLEAs). The results in Figure 5 present the percentage of CLEAs residual activity during the storage at 4°C for 44 days. The activity of CLEAs was compared with the results right before synthesis, so this value was considered as 100% activity. Indeed, the residual activity of CLEAs and mCLEAs gradually decreased depending on the number of days, whereby TGM CLEAs were found to be the most stable, as their activity was still 43% after 35 days. It kept a 100% activity during the first 4 days, and later a slight activity decrease was detected. Almost no significant differences in residual activity of β-gal CLEAs during the first 4 days of storage at 4°C was detected. However, the half-life of CLEAs was achieved after 16th day of storage at 4°C. In addition, even after 44 days of storage at 4°C, the β-gal CLEAs were still active with a residual activity of 20%. According to research from Xu and co-workers, β-gal CLEA retained 90% activity after 2 weeks of storage at 4°C in buffer (Xu et al., 2020), which is comparable to the results of our study. In this context, Liao and co-workers described the storage stability of halohydrin dehalogenase CLEAs, which maintained approximately 23% residual activity after incubating for 2 months at 4°C (Liao et al., 2018).

Figure 6 shows the percentage of mCLEAs residual activity during the storage at 4°C for 44 days, assuming that the activity on the first day was 100%. A loss of activity from an initial 100% to 19% for laccase mCLEAs after 44 days of storage at 4°C was observed. Compared to the laccase CLEAs stability, this was the same for mCLEAs. Moreover, the residual activity of β-gal mCLEAs decreased with prolonged storage time at 4°C. It is evident that the residual activity remained high after the first 8 days of storage at 4°C, resulting in almost 90% of its initial activity. On the 20th day, the residual activity of β-gal mCLEAs decreased to 55%, and on day 29, the β-gal mCLEAs retained the residual activity of 32%, which is comparable to β-gal CLEAs stability. The half-life of β-gal mCLEAs was achieved after the 20th day of storage at 4°C, which is 4 days longer than for β-gal CLEAs.

The storage stability was also preserved better by cellulase mCLEAs, compared to cellulase CLEAs, which reached half-life *t*½ on the 13th day, while cellulase mCLEAs showed 64% activity barely after 22 days. This proves that when the enzymes are immobilized as mCLEAs, a large number of multiple interactions were formed between enzyme–enzyme and enzyme–MNP. These bonds provide a confinement effect and a more effective conformational stabilization of protein structure. The findings from Dal Magro and co-workers are in agreement with ours, as their work described that cellulase mCLEAs are less sensitive to storage inactivation (Dal Magro et al., 2018). Several studies point out the positive effects on the stabilization of enzymes by immobilization *via* CLEAs and mCLEAs, which, however, decreases over time (Cui et al., 2014; Xu et al., 2020).

These data clearly demonstrate a continuous loss of mCLEAs activity over time, but in comparison to the stability of CLEAs, the mCLEAs show slightly better stability at storage conditions.

When the activity of TGM CLEAs and TGM mCLEAs during storage are compared, it was observed that the stability of TGM mCLEAs was much higher, as after 8 days, a decrease in activity to 80% in CLEAs was noticed, while TGM mCLEAs after 8 days showed 86% residual activity. Moreover, mCLEA was much more stable compared to TGM CLEAs. The involvement of polypeptides of TGM in these intermolecular reactions can result in the formation of a large crosslinked protein network (Schmid et al., 2014). For this reason, TGMs render well-maintained stability, which is important for the application of this enzyme. Heretofore, there are no reports on crosslinked TGM aggregates produced up to now. Therefore, this work provides data on the stability of the immobilized TGM.

According to the statistical analysis of the results, paired *t*-test confirmed that residual activity of mCLEAs was significantly higher than that of CLEAs [*t*(14) = 2.76, *p* = 0.008]. The mean residual activity of mCLEAs in the second week was 76.25% ± 10.86%, while the mean residual activity of CLEAs was slightly lower, 67.62% ± 19.02%. In the final week, mCLEAs showed 35.5% ± 23.33% of residual activity, while the mean residual activity of CLEAs was 27.5% ± 10.61%. Additionally, ANOVA confirmed statistical differences between the type of enzymes (*F* = 12.52, *p* < 0.001), and Tukey multiple comparisons of means confirmed that transglutaminase was statistically different (*p* < 0.05) to all other enzyme groups.

In general, multipoint covalent binding is one of the strongest chemical bonds used to immobilize enzymes. The formation of multiple covalent bonds between the enzyme and the carrier reduces conformational flexibility and fixes the enzyme, which prevents protein unfolding and denaturation. Furthermore, multipoint covalent attachment of enzymes on highly activated pre-existing supports *via* short spacer arms and involving many residues placed on the enzyme surface promotes a rigidification of the enzyme structure of the immobilized enzyme (Cui et al., 2018), which consequently results in prolonged stability of mCLEA.

### 3.5 Recycling Stability of CLEAs and mCLEAs

**FIGURE 7:**
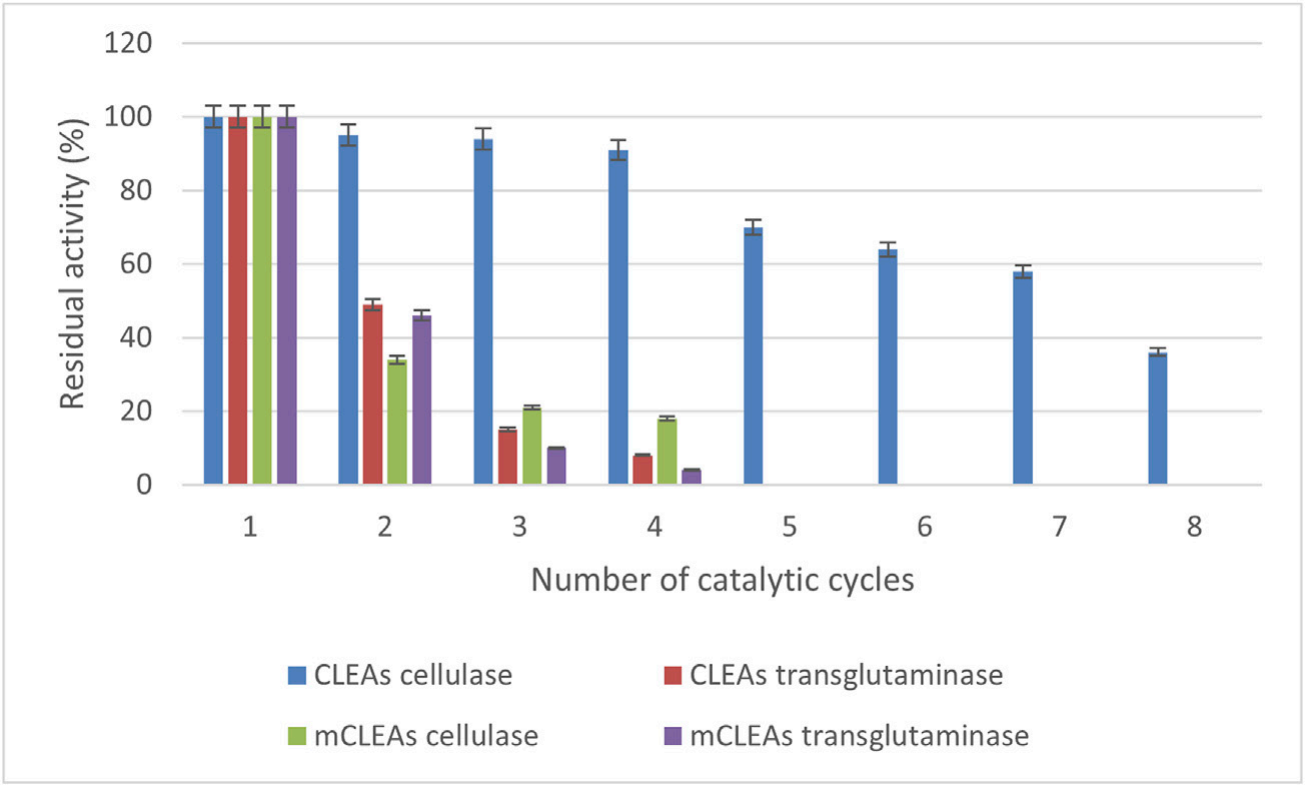
The CLEAs and mCLEAs reusability depend on the number of cycles.

The reusability of enzymes is an important criterion in industrial applications for making the processes cost-effective and efficient in downstream operations (Han et al., 2020). Therefore, the stability of immobilized CLEAs and mCLEAs was checked by determining residual activity after several consecutive cycles. This study investigated the reusability of CLEAs and mCLEAs prepared from cellulase and transglutaminase. In principle, the activity of CLEAs and mCLEAs just after preparation was taken as the initial 100% activity. Results in Figure 7 demonstrated that CLEAs cellulase retained as high as 50% of initial activity after the seventh usage, whereas mCLEAs cellulase retained only 18% of its original activity after the fourth consecutive cycle. In contrast, it was found that TGM CLEAs retained 49% of initial activity after the second catalytic cycle, and a significant activity decrease (almost 90% in third cycle) was observed. Hence, more than 50% of the initial activity of TGM mCLEAs was lost after the second cycle and did not show any activity after just 5 times of usage.

In the current study, the enzymatic activity declined probably because of mechanical losses of CLEAs and mCLEAs during the washing and centrifugation procedures, which led to a loss of aggregates that could not be recovered. This was especially true for TGM CLEAs and mCLEAs. Although it had long-term storage stability at 4°C, the reusability was not as high as CLEAs and mCLEAs from cellulase.

The results of our study are encouraging compared to the published study of Perzon and co-workers, which reported reusability conditions of the polyethylene glycol-CLEAs from cellulase with 40% of its initial activity recovered after four consecutive runs, and the ammonium sulfate-CLEAs from cellulase with only 10% of its initial activity recovered after just one use (Perzon et al., 2017). Hence, these results highlight the fact that CLEAs from cellulase exhibit good reusability, which presents important aspects in further studies and applied sciences.

## 4 Conclusion

Generally, immobilized enzymes exhibited higher tolerance than free enzymes under certain conditions, which usually cause denaturation of the enzyme. In the present study, the versatility and main strategy used for the preparation of immobilized biocatalysts as CLEAs and mCLEAs were demonstrated. During this research, it was found that the optimal precipitation reagents for obtaining the highest residual activity of the resuspended enzyme in the synthesis of CLEAs and mCLEAs were ethanol for cellulase CLEAs, 1-propanol for β-gal and laccase CLEAs, and 2-propanol for TGM CLEAs, with 84%, 102%, 65%, and 231% of residual activity, respectively. After successful enzyme precipitation, optimization of various process conditions, the concentration of GA, NaBH_3_CN, and enzyme, and the highest relative activity of CLEAs and mCLEAs from different enzymes were obtained. The highest relative activities, 118% and 103%, were obtained in β-gal CLEAs and β-gal mCLEAs, respectively. Cellulase CLEAs and TGM mCLEAs expressed 94% and 73% relative activity, respectively. The lowest relative activity of 30% was obtained in laccase mCLEAs, which, however, showed good storage stability and retained 20% residual activity even after 20 days. Certainly, the highest residual activity of 52% after storage conditions showed TGM mCLEAs after 44 days of storage at 4°C. Statistical analysis showed better storage stability of mCLEAs compared to CLEAs. Among other things, the stability of CLEAs and mCLEAs in scCO_2_ was found to be useful for enzymatic catalysis in scCO_2_, where TGM CLEAs and laccase mCLEAs achieved hyperactivation of 123% and 105% after the exposure to scCO_2_ conditions at 10 MPa, compared to the unexposed CLEAs and mCLEAs. As investigated, the residual activity of CLEAs and mCLEAs decreased with increased pressure and time. In comparison to mCLEAs, in the most cases, the CLEAs show better stability in scCO_2_ conditions. This can be due to the suitable microenvironment and constrained steric structure of CLEAs and depends on the presence of magnetic nanoparticles in mCLEAs, which can lead to significant changes in enzyme molecular structure. Finally, the whole study was devoted to the preparation of two different immobilization techniques, CLEAs and mCLEAs, and a comparative analysis of four different enzymes with their relative activity and stability under different conditions was also performed. No common rule about optimal immobilization parameters for all enzymes can be predicted. In general terms, this research suggested that the immobilization of different enzymes in CLEAs and mCLEAs improves the stability and could be promisingly employed in different bioapplications.

## Data Availability

The raw data supporting the conclusion of this article will be made available by the authors, without undue reservation.
