# Supplementary material for: The Synthesis of (Magnetic) Crosslinked Enzyme Aggregates With Laccase, Cellulase, β-Galactosidase and Transglutaminase
Source: Front Bioeng Biotechnol. 2022 Mar 3;10:813919. doi: 10.3389/fbioe.2022.813919 (PMC8927696; doi:10.3389/fbioe.2022.813919)
Supplement: Supplementary file 1 [file DataSheet1.docx]

Supplementary Material

# Testing the stability of CLEAs and mCLEAs in scCO_2_

The stability of CLEAs and mCLEAs were tested in the high-pressure batch reactor on different pre-set temperature for specified exposure time. A schematic diagram of the experimental setup used in this study is shown in the Supplementary Figure 1, where the high-pressure reactor was temperature- and pressure-regulated with a volume of 60 mL.


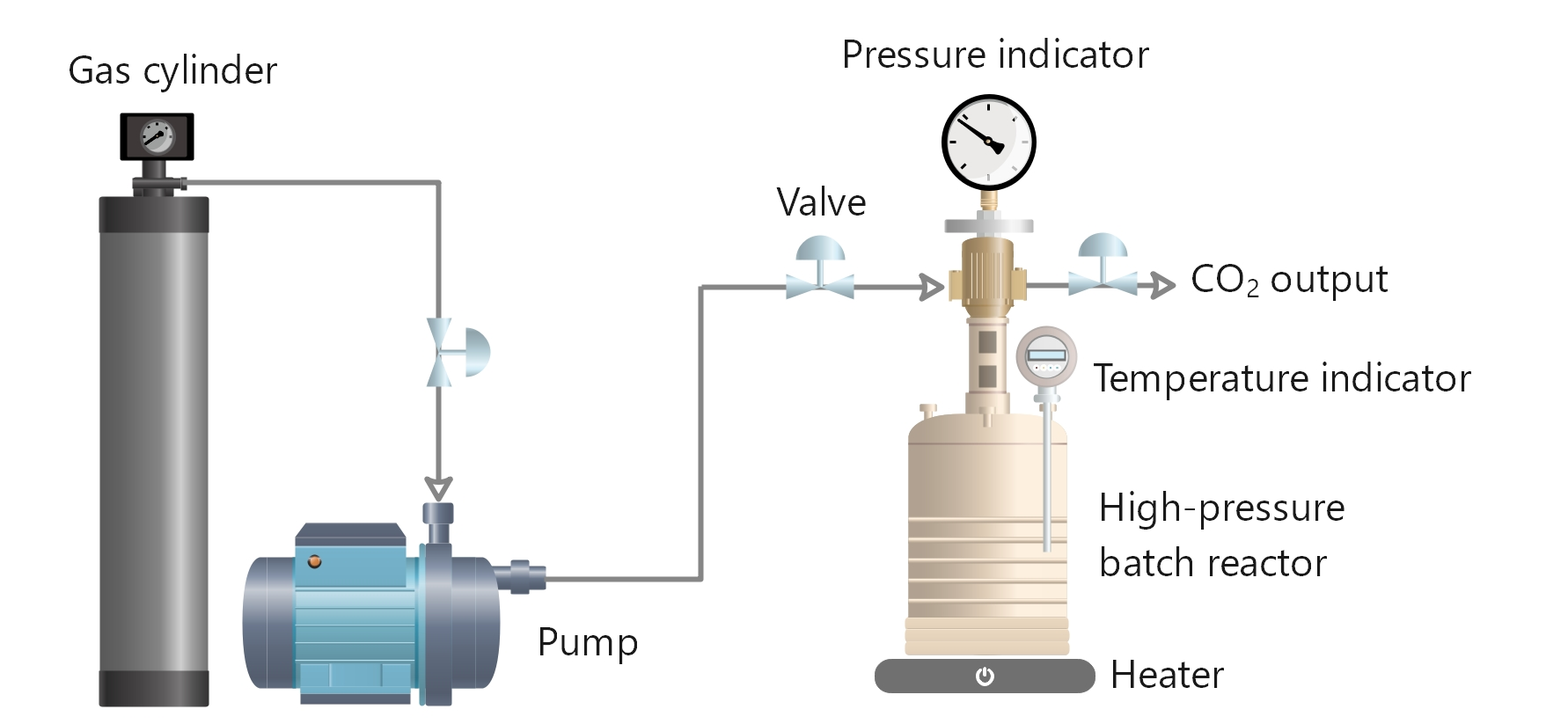


**Supplementary Figure 1.** Schematic diagram of the experimental setup for scCO_2_ treatment with units description.
